# The White Matter of Aha! Moments

**DOI:** 10.21203/rs.3.rs-6658726/v1

**Published:** 2025-05-22

**Authors:** Carola Salvi, Simone A. Luchini, Franco Pestilli, Sandra Hanekamp, Thomas Hope, Todd Parrish, Mark Beeman, Jordan Grafman

## Abstract

Insights, or "Aha!" moments, are a crucial aspect of idea generation in creative cognition. While functional neuroimaging studies have identified brain regions involved in these insights, the white matter substrate of insights remains unexplored. This study employed Diffusion Tensor Imaging (DTI) to investigate how white matter microstructure—measured by Fractional Anisotropy (FA) and Mean Diffusivity (MD)—relates to individuals’ tendency to solve Compound Remote Associates problems through insight *versus* step-by-step analytical reasoning. After controlling for age and gender, insightfulness was found to be associated with lower FA (and higher MD) in the left posterior Arcuate Fasciculus (AF) and bilateral Superior Longitudinal Fasciculi III. Conversely, step-by-step idea generation was linked to higher FA (and lower MD) in the left Vertical Occipital Fasciculus (VOF) and to higher FA in the anterior corpus callosum. These findings suggest that insight may benefit from more diffuse connectivity patterns, allowing for broader semantic activation and cognitive flexibility, while analytical idea generation relies on stronger structural connections supporting executive control. Our study provides novel evidence for distinct structural connectivity patterns associated with different idea-generation approaches, contributing to a more comprehensive understanding of the neural architecture supporting creative cognition.

## 1. Introduction

Idea generation plays a crucial role in driving human innovation, from scientific discoveries to artistic breakthroughs. Scientists have identified two main ways people generate creative ideas and solve problems: through sudden insights or a continuous step-by-step "analytical" process (Jung-Beeman et al., 2004; Kounios & Beeman, 2014; Salvi, Wiley & Smith, 2024). Insights are characterized by an unexpected discovery or transformative idea (Csikszentmihalyi & Sawyer, 1995; Simonton, 1999) which emerges into awareness suddenly, in a discontinuous manner, often interrupting one's train of thoughts (Smith & Kounios, 1996; Salvi, 2023). By contrast, analytical ideas are yielded by a deliberate and controlled process. Insights are accompanied by a subjective "Aha!" experience and they entail a conceptual restructuring that results in a novel, non-obvious interpretation of people's mindset, which is often identified as a form of creativity (Friedman & Förster, 2005, Salvi, 2023). Insightful ideas have been demonstrated to be more accurate and creative than deliberate step-by-step solutions since they rely on information that may appear distantly related to the original problem and on the retrieval of uncommon interpretations of problem elements (Kounios & Beeman 2014; Salvi et al., 2016; Danek & Salvi, 2018). This is partly because insight entails below-awareness recombination of information, allowing for the formation of novel associations that emerge into consciousness suddenly and often without warning. This subjective quality of suddenness is thought to be distinct from the more accumulative results that emerge from deliberative reasoning (Bowden & Beeman, 1998; Bowden et al., 2005; Danek & Salvi, 2018; Laukkonen, 2024; Salvi et al., 2016; Schooler & Melcher, 1995; Smith & Kounios, 1996). While both insight and analytically derived ideas play a role in creative cognition and problem-solving, their phenomenology and underlying brain circuitries are different (Chesebrough et al., 2024; Danek et al., 2024; Jung-Beeman et al., 2004; Kounios & Beeman, 2014; Salvi, 2023; Salvi & Bowden, 2024). While functional neuroimaging research has elucidated the brain mechanisms underlying insight-based idea generation (for comprehensive reviews, see Chesebrough et al., 2024; Kounios & Beeman, 2014; Salvi 2023; Salvi & Bowden, 2024) a growing body of research indicates that structural brain connectivity patterns may correlate uniquely with different cognitive functions (Salvi, Wiley & Smith; 2024). Nonetheless, research investigating the link between white matter connectivity and individual differences in insight propensity remains elusive. Diffusion Tensor Imaging (DTI) offers a unique opportunity to address this knowledge gap by providing detailed information about white matter microstructure and connectivity patterns in the brain (Basser et al., 1994; Mori & Zhang, 2006).

DTI is a magnetic resonance imaging technique that measures the diffusion of water molecules in biological tissues, particularly in white matter tracts (Beaulieu, 2002; Mori et al., 2005). This method allows researchers to visualize and quantify the organization and integrity of white matter fibers, providing insights into the structural connectivity between different brain regions (Kreher et al., 2008; Le Bihan et al., 2001). By analyzing the direction and magnitude of water diffusion DTI can reveal the orientation and properties of white matter pathways, offering a non-invasive means to study brain structure *in vivo* (Assaf & Pasternak, 2008). The application of DTI to investigate insight-related individual differences is crucial for several reasons. First, it can provide complementary structural information to existing functional studies, revealing the underlying white matter pathways that facilitate communication between regions activated during insight (Jones et al., 2013). Second, DTI allows for the examination of stable, trait-like structural characteristics that may predispose individuals to experience insights more frequently (Johansen-Berg, 2010). By mapping white matter tracts, DTI can elucidate how different brain regions involved in insight are structurally connected, potentially revealing integrated networks supporting this cognitive process (Bullmore & Sporns, 2009). Furthermore, DTI metrics such as fractional anisotropy (FA) and mean diffusivity (MD) can quantify individual differences in white matter integrity and provide information about the average molecular motion of water in brain tissue (Nestor et al., 2004; Wahl et al., 2010), potentially correlating with behavioral measures of insight propensity. Thus, a DTI study investigating individual differences in insight problem solving would significantly advance our understanding of the structural neural substrates underlying this critical aspect of creative cognition, complementing existing functional neuroimaging findings and providing a more comprehensive picture of the neural basis of insight.

#### 1.1 DTI Literature on Convergent and Divergent Thinking

Traditionally, scientific literature in the field of creativity distinguishes between convergent and divergent thinking (Guilford, 1968). Convergent thinking involves focusing on a single correct solution to a problem, whereas divergent thinking is characterized by the generation of multiple, diverse, and original solutions to open-ended problems (Runco & Acar, 2012). While both these processes contribute to creativity, they engage different cognitive mechanisms and neural pathways (e.g., Fink et al., 2007). Applications of DTI to the study of creativity have yielded inconsistent findings, reflecting the complexity of creative cognition and the challenges in its measurement. Some studies have reported lower FA across several white matter tracts (Jung et al., 2010; Ryman et al., 2014; Wertz et al., 2020), while others have observed the opposite relationship (Rahmani et al., 2020; Takeuchi et al., 2010, 2020; Wu et al., 2021; Zhang et al., 2022). These inconsistencies may be attributed to variations in task administration and the potential conflation of divergent and convergent thinking processes. The main results are outlined below.

*Divergent Thinking*

The most common divergent thinking measure is the Alternate Uses Task (AUT), which involves the generation of unusual uses for everyday objects (Guildford, 1967). Seminal investigations into the structural brain underpinnings of divergent thinking revealed intriguing patterns. Jung et al. (2010) observed a negative correlation between FA within left inferior frontal white matter and performance on the AUT.

Wertz et al. (2020) extended these findings, reporting negative correlations between FA and divergent thinking abilities across a series of predominantly left-lateralized tracts spanning both frontal and temporal regions. This study employed a composite score derived from multiple creativity tasks, not limited to the AUT, which may account for the broader range of implicated tracts.

By contrast, other studies then reported positive associations between FA across several bilateral white matter tracts and divergent thinking abilities (Rahmani et al., 2020; Takeuchi et al., 2010; Zhang et al., 2022). For example, Takeuchi et al. (2010) found positive correlations between FA and divergent thinking abilities in regions including the bilateral prefrontal cortices, corpus callosum (CC), bilateral basal ganglia, bilateral temporoparietal junctions (TPJ), and right inferior parietal lobule, suggesting enhanced structural connectivity in these areas may support creative cognitive processes.

The inconsistencies in these results could reflect variations in the way authors measured either divergent thinking, DTI, or both. First, the studies did not follow a single, standardized DTI pre-processing pipeline, and different pipelines can yield different results (Maier-Hein et al., 2017). Second, few studies measured divergent thinking using the same task (or collection of tasks); there was also variation in how "creativity" scores were then derived from task scores. Third, this methodological variation is likely compounded by demographic variation across the samples employed in these studies, with sample features such as gender balance, and linguistic and cultural background already known to mediate divergent thinking (Wertz et al., 2020).

*Convergent Thinking*

One study reported a positive correlation between performance on the German version of the Remote Associates Task[1] and FA across the right corticostriatal pathway (Rahmani et al., 2020). Whereas another study reported significant positive correlations between convergent thinking abilities and FA across the left inferior longitudinal fasciculus (ILF) and the left frontal-occipital fasciculus (FOF) as well as the CC (Takeuchi et al., 2020). In this latter case, researchers employed a Japanese variant of the CRA (JRAT), which varies from the English and German versions given linguistic differences between the two languages, and variability in the task instructions. For instance, the JRAT requires participants to replace linguistic units of writing (i.e., kanjis) from the prompt words, while the English and German versions do not involve any replacement. Crucially, none of these studies investigate idea generation via insight. Thus, is it impossible to draw reliable conclusions on individual differences in white matter structure related to insight.

In sum, while past findings collectively suggest a relationship between white matter structure and creative thinking, these findings still lack a clear and reliable explanation of the precise nature of this relationship. Additionally, they do not directly address the specific aspect of creativity that our study focuses on: the role of Aha! Moments in creative problem solving.

#### 1.2 Insight and Convergent Thinking

Insight is often studied using convergent thinking tasks such as the Remote Associates (RAT or Compound Remote Associates - CRA) problems because of their methodological advantages and statistical power (Bowden & Beeman, 2003; Salvi, Costantini, et al., 2015, Salvi, 2023). Remote Associates are compact, quick to solve, and elicit both insight and step-by-step analytical solutions, allowing for efficient experimental designs and direct comparisons between problem-solving modes. Their verbal format and reliance on semantic processing make them well-suited for neuroscientific research into the neural correlates of insight (Jung-Beeman et al., 2004; Kounios et al., 2006, Salvi et al., 2020; Salvi, 2023).

#### 1.3 Neural Correlates of Insight

The pioneering study that delved into the neural basis of insight employed both fMRI and high-density EEG in separate experiments with a consistent methodology (Jung-Beeman et al., 2004). Among several results (see Kounios & Beeman, 2014 for a review), the researchers found a specific localized neural activity associated with the Aha! Moment over the right temporal cortex. Their EEG results showed a sudden burst of 40-Hertz gamma-band activity over this brain region, occurring 300 milliseconds before participants pressed a button to signal their insight. Imaging results pinpointed this activity to the medial aspect of the right Superior Temporal Gyrus (STG). This brain area is known for its role in semantic integration of distantly related associations necessary for achieving global coherence in reasoning and discourse processing (St. George et al., 1999), as well as understanding novel metaphoric expressions, implicit comprehension, and humor (Bartolo et al., 2006; Goel & Dolan, 2001; Manfredi et al., 2017; Mashal et al., 2007; Wakusawa et al., 2007).

Researchers have argued that the right STG supports the connection of distantly related information during insight, enabling solvers to perceive associations that would otherwise be missed (Bowden & Jung-Beeman, 2003; Jung-Beeman et al., 2004). Subsequent studies using various brain stimulation techniques, such as transcranial Alternating Current Stimulation and transcranial Direct Current Stimulation, provided causal evidence for the role of the right temporal lobe in insight problem solving (Chi & Snyder, 2011, 2012; Salvi et al., 2020; Santarnecchi et al., 2019; Shen et al., 2017; Sprugnoli et al., 2021). Conversely, the left temporal lobe appears to support finer semantic coding, characterized by more focused neural activity leading to one or a few dominant interpretations or alternative meanings (Beeman et al., 1992; Chiarello et al., 1990; Jung-Beeman, 2005; Koivisto, 1997). This region tends to be "chronically inhibited" in individuals who solve problems via insight, perhaps promoting the emergence of weakly activated information processed in the right temporal lobe (Erickson et al., 2018).

Early studies on the neuroscience of insight discovered that gamma-band oscillation over the right temporal lobe is preceded by alpha-band activity over the right occipital cortex which is thought to reflect the inhibition of visual inputs (Jung- Beeman et al. 2004; Jensen & Mazaheri, 2010; Jung- Beeman et al., 2004). More recently, Yu and colleagues (2025) found that more time spent in a state with widespread alpha-band synchronization was associated with generating more novel metaphors. This aligns with studies showing increased alpha power during tasks requiring the generation of novel uses for common objects (Agnoli et al., 2020; Di Bernardi Luft et al., 2018; Jauk et al., 2012; Stevens & Zabelina, 2020), novel names for abbreviations (Fink et al., 2009), and explanations for unusual situations (Grabner et al., 2007).

Alpha-band synchronization is thought to facilitate selective inhibition (Klimesch et al., 2007) and is sensitive to internal processing demands such as during creative thinking (Jensen & Tesche, 2002). In the context of insight problem solving, inhibition plays a crucial role in suppressing dominant but unhelpful interpretations, allowing less obvious associations to surface. Alpha oscillations, particularly in parietal-occipital and prefrontal regions, have been linked to this inhibitory process, helping individuals disengage from irrelevant cognitive processes and shift toward novel, insightful solutions (Klimesch et al., 2007; Sauseng et al., 2005). This mechanism aligns with findings that insight often requires overcoming habitual thought patterns and permitting weakly activated remote associations—processed in the right hemisphere—to gain prominence (Kounios & Beeman 2014; Salvi, 2023).

FA has been found to correlate with the alpha rhythm, suggesting a structural basis for individual differences in oscillatory dynamics (Valdés-Hernández et al., 2010). Specifically, research indicates that FA, which reflects white matter integrity, fiber density, and myelination, is significantly related to the alpha peak frequency, with stronger correlations observed in posterior commissural fibers and thalamocortical pathways dynamics (Valdés-Hernández et al., 2010). This relationship likely stems from the role of white matter in facilitating communication between distant brain regions. Given that alpha-band activity is thought to support inhibition and top-down control processes, variations in FA could influence the efficiency of these mechanisms. For instance, higher FA in the superior and posterior Corona Radiata, which are implicated in thalamocortical interactions, has been linked to increased alpha frequency, potentially reflecting more effective inhibition of irrelevant sensory information. Conversely, lower FA in certain regions may contribute to weaker inhibitory control, leading to reduced alpha synchronization (Valdés-Hernández et al., 2010).

In summary, past work illustrates the role of lateralized functional patterns in supporting insight problem solving (for a review, see Salvi, 2023), while emerging research highlights the importance of selective inhibition in facilitating the cognitive flexibility needed to reach insightful solutions. While relationships between white matter integrity and alpha band activity provide some initial evidence for the role of brain structures in insight, no work to our knowledge has investigated the possible link between white matter and insightful problem solving.

#### 1.4 The Present Research

Given the existing literature on insight and creative cognition, as well as the inconsistent findings in DTI studies in the field, we decided to investigate the structural connectivity patterns associated with individual differences in insight propensity. We thus employed DTI to examine white matter microstructure and connectivity patterns in relation to insight and analytical problem solving on the CRA.

Based on previous functional neuroimaging and brain stimulation studies, we generated several compatible hypotheses: (1) Individuals with higher insight propensity would show lower FA values in left hemisphere white matter tracts, particularly those connected to the left temporal lobe. This prediction is based on the work of Jung-Beeman and colleagues (2004, 2005) and Erickson et al., (2018), which suggests that inhibition of the left temporal lobe may promote the emergence of weakly activated information processed in the right temporal lobe, facilitating insight. (2) Convergent thinking would be linked to higher FA values in the CC, similar to the findings of Takeuchi et al. (2010, 2020). However, our study will extend these findings by specifically examining whether these structural differences are associated with insight or step-by-step analytical solutions to the CRA problem, which could have been a confound of prior results. Unlike previous studies that did not differentiate between problem-solving modes, our approach will allow us to determine if the increased FA values are specifically related to insight processing or if they reflect more general problem-solving abilities. This distinction is crucial for understanding the unique structural correlates of insight as opposed to non-insight solving.

By testing these hypotheses, our study aims to bridge the gap between functional and structural neuroimaging findings in insight research, potentially revealing the underlying white matter architecture that supports individual differences in insight propensity. Further, we employ a pre-processing DTI pipeline using the open-source brainlife.io platform, which ensures straightforward replicability of our methodology without the need for custom code. This investigation will contribute to a more comprehensive understanding of the neural basis of insight, complementing existing functional studies and shedding light on the stable, trait-like characteristics that may predispose individuals to experience insights more frequently.

[1] The Remote Associates Task or RAT is a variant of the CRA, which instead involves finding a single word that is semantically related to three prompt words.

### 2. Methods

#### 2.1 Subjects

44 right-handed, native American English speakers were recruited for the study. Participants were eligible for the study if they met the following criteria: (1) no history of neurological or psychiatric disorder; (2) no use of central nervous system or mood and attention-affecting drugs (such as antidepressants, amphetamines, or anxiety medications); and (3) no history of traumatic brain injury or intracranial metal implantation. Participants older than 45 years old (3 participants) and those who solved less than 10 problems (2 participants) or no problems (either via insight or via step-by-step analysis) because they misunderstood the instructions (1 participant) were excluded from the analysis. Thus, a final sample of 38 participants (25 self-identified as females; 13 self-identified males; M age = 24.55; SD = 5.2) was used for the data analysis. Participants' level of education corresponded to an average of 15.8 years (SD = 3.1). Participants were paid for completing the study and each experimental session lasted approximately 1.5 hours. The study was approved by the Northwestern University Institutional Review Board, and all participants gave written informed consent.

#### 2.2 Behavioral Measures of Problem Solving

Participants were presented with 60 difficulty-balanced CRA problems, selected from the item pool developed by Bowden and Jung-Beeman (2003), and randomized in their order of presentation. Each trial consisted of three stimulus words (e.g., *crab*, *pine*, and *sauce*), and participants were tasked with identifying a fourth word that could form a common compound word or two-word saying with each of the given words (e.g., *apple*) within a 15-second time limit. After each problem, participants were required to self-report whether they achieved the correct solution via insight or step-by-step analysis. Self-reporting of insightful experiences has been established as accurate and reliable based on numerous behavioral and neuroimaging studies (e.g., Bowden & Jung-Beeman, 2007; Jung-Beeman et al., 2004; Salvi, et al., 2015; Salvi, Beeman, Bikson, McKinley, & Grafman, 2020).

CRA problems featured words displayed in 28-point Times New Roman font, presented in black color text on a white background, and centrally aligned. The experiment was conducted using E-Prime 2.10 software, presented on a 24-inch Dell screen with participants viewing from approximately 60 cm away. During the instructional phase, participants were trained to distinguish between insight and step-by-step analytical problem-solving approaches when tackling a problem. [2] Over the past two decades, CRA problems have emerged as a well-established tool for investigating insight problem-solving and their neural corelates, as they encompass the fundamental characteristics of traditional insight tasks with higher solving reliability and statistical power (Bowden & Beeman, 2006; Jung-Beeman et al., 2004; Salvi; 2023). Additionally, success in solving CRAs has been found to correlate with success in solving classic insight problems (Ball & Stevens, 2009; Bowden et al., 2005; Dominowski & Dallob, 1995; Schooler & Melcher, 1995).

#### 2.3 Image acquisition

Participants underwent MR imaging using a Siemens 3T Prisma Fit (sw version VE11C) scanner with a 64-channel head coil at the Center for Translational Imaging, Northwestern University. A navigated, multiecho MPRAGE 3D T1-weighted sagittal volume was collected (TR/TE1/TE2/TE3 = 2170/1.69/3.55/5.41 ms, TI = 1160 ms, flip angle = 7∘, FOV = 256x256 mm2, voxel size = 1x1x1 mm3, GRAPPA inplane acceleration=2, 176 sagittal slices). A root mean square volume was generated from the 3 TE volumes to maximize image quality. A multi-shell, multi-band high-resolution DTI sequence (TR=3000 ms, TE = 72.4 ms, flip angle = 90∘, FOV = 222x222 mm2, voxel size = 1.5x1.5x1.5 mm3, multiband factor=4, GRAPPA inplane acceleration=2, 96 interleaved slices, phase encode direction A>P) with 64 gradient directions per shell (b values = 1000, 2000 s/mm2, and 1 non-diffusion weighted (b = 0) volumes was acquired in the axial plane.

### 2.4 Data pre-processing

Anatomical processing. T1-weighted anatomical images preprocessed and aligned to the anterior commissure–posterior commissure (ACPC) plane using brainlife.app.273. Following alignment, the ACPC-aligned T1w anatomical scans for each participant were segmented to generate 5-tissue type (5tt) masks using functionality provided by *MRTrix3* (Tournier et al, 2019) implemented as brainlife.app.239. The resulting 5tt masks were then used as seed masks for subsequent white matter tractography. Additionally, the aligned anatomical T1w images were used to segment and generate surfaces using Freesurfer 7.1.1's *recon-all* function (Fischl, 2012) (brainlife.app.462).

### 2.5 Diffusion (dMRI) processing

*Preprocessing & model fitting:* Diffusion MRI (dMRI) data underwent preprocessing as the protocol outlined in (Ades-Aron et al., 2018) using brainlife.app.68. The preprocessing pipeline began with denoising and removal of Gibbs ringing artifacts using *MRTrix3* functions before being corrected for susceptibility, motion, and eddy distortions and artifacts via FSL's *topup* and *eddy* functions (Andersson et al., 2003; Smith et al., 2004). Eddy-current and motion correction utilized FSL's *eddy_cuda8.0* with the replacement of outlier slices (*i.e., repol*) command(Andersson et al., 2016, 2017, 2018; Andersson & Sotiropoulos, 2016). To address potential misaligned gradient vectors, MRTrix3's *dwigradcheck* functionality was used (Jeurissen et al., 2014). Further steps involved debiasing using ANT's *n4* functionality (Tustison et al., 2014) and background noise removal using MRTrix3.0's *dwidenoise* functionality (Veraart et al., 2016). Finally, the preprocessed dMRI images were registered to the anatomical (T1w) image using FSL's *epi_reg* functionality (Greve & Fischl, 2009; Jenkinson et al., 2002; Jenkinson & Smith, 2001). After preprocessing, the diffusion tensor (DTI) model (Pierpoli et al, 1996) model was fit to the preprocessed dMRI images for each subject using brainlife app brainlife.app.319 for DTI model fitting.

*Whole-brain tractography:* Whole-brain tractography was performed using anatomically-constrained probabilistic tractography (ACT; Smith et al., 2012) as implemented in MRtrix3 via the brainlife.io app (brainlife.app.319). Fiber orientation distributions were estimated following model fitting, and tractography was seeded throughout the white matter. Tracking parameters were set to a step size of 0.2 mm, with minimum and maximum streamline lengths of 20 mm and 220 mm, respectively. The maximum curvature angle between successive steps was limited to 35°. Anatomical constraints were applied using the 5TT segmentation to ensure that streamlines were biologically plausible, initiating and terminating in the gray matter and remaining within white matter pathways.

*Segmentation of white matter tracts:* After whole-brain tractography was performed, 61 major white matter tracts were segmented for each run using a customized version of the white matter query language (Bullock et al, 2019) implemented as brainlife.io app brainlife.app.188. Outlier streamlines were subsequently removed using functionality provided by Vistasoft implemented as brainlife.io app brainlife.app.195.

*Tract Profile Analysis:* Following cleaning, tract profiles with 200 nodes were generated for all DTI measures across the 61 tracts for each subject and test-retest condition using functionality provided by Vistasoft implemented as brainlife.io app brainlife.app.361. In order to avoid partial-voluming effects and cleanly separate the bundle from gray matter, we removed the first and last 10 nodes from the tract profiles using brainlife.app.685. Average FA and MD values were then computed for each tract using brainlife.app.706, along the 180 nodes, and used for further analysis. (See supplementary material for brainlife.app details).

[2] Specifically, the following instructions were given to participants to explain how to distinguish a solution via insight from one via analysis: *You will decide whether the solution was reached with insight or with analysis. With INSIGHT means you experienced a so-called A-ha! moment and the solution came to mind as a sudden surprise. It won't be a huge Eureka, just a small surprise and it may be difficult to articulate how you reached the solution. STEP-BY-STEP it means that you reached the solution gradually, part by part. You might have used a deliberate strategy or just trial-and-error and you can report steps. We know it is not always obvious whether you used insight or step-by-step, and you may feel as though you used a mixture of both. But we need you to choose one the best you can, so please choose whichever method your solving process most closely resembles. No solution type is better or worse than the other; there are no right or wrong answers in reporting insight or analysis.* Instructions used were similar to those used by Bowden and Jung-Beeman 2003.

## 3. Results

Behavioral data analysis was performed using JASP version 0.18.1 (JASP Team - 2023) and the significance level was set to $p < 0.05$. Data were tested for normality (Kolmogorov–Smirnov test) and homogeneity of variance (Levene's test). Data were normally distributed and assumptions for the use of analysis of variance were not violated.

In DTI analysis, converging findings from FA and MD provide validity to any underlying white matter microstructural differences. FA is a measure of the directional coherence of water diffusion, reflecting the degree of myelination and axonal integrity within a white matter tract. MD, on the other hand, reflects the overall magnitude of water diffusion, providing information about tissue density and membrane permeability. When the observed effects for FA and MD are in the expected opposing direction, it strengthens any interpretations linking structural connectivity patterns to cognitive processes, rather than these being driven by non-specific factors. In our data, the concordance between these two complementary DTI metrics lends greater confidence to our conclusions regarding the relationship between white matter microstructure and individual differences in insight versus analytical problem solving.

#### 3.1 Problem solving

Out of 60 CRA problems participants correctly solved an average of 23.42 problems (SD = 7.2) per person[3]. Of the 60 administered, an average of 12.8 (SD = 5.9) problems per person were correctly solved via insight, and an average of 10.6, (SD = 10.7) problems per person were solved correctly via step-by-step analysis. Of the 60 administered an average of 6.5 (SD = 7.2) were commission errors, 2.8 (SD = 4.2) by insights, and 3.65 (SD = 4.1) by step-by-step. These solution averages are consistent with prior findings (Chein & Weisberg, 2014; Cranford & Moss, 2013; Kounios et al., 2006; Salvi et al., 2015, 2016; Salvi & Bowden, 2019; Stuyck et al., 2021, 2022). An overall correlation analysis between the number of solutions via insight and step-by-step, whole brain FA, and MD is reported in Table 1.

*Table 1. Correlation Matrix between the number of problems solved via insight and step-by-step analysis, whole brain FA, and MD.*

| Variable | | 1 | 2 | 3 | 4 |
|---|---|---|---|---|---|
| 1. Insight | Pearson's r | — | | | |
| | p-value | — | | | |
| | Effect size (Fisher's z) | — | | | |
| | SE Effect size | — | | | |
| 2. Step-by-step | Pearson's r | -0.247 *** | — | | |
| | p-value | < .001 | — | | |
| | Effect size (Fisher's z) | -0.253 | — | | |
| | SE Effect size | 0.015 | — | | |
| 3. FA | Pearson's r | -0.045 ** | -0.009 | — | |
| | p-value | 0.002 | 0.541 | — | |
| | Effect size (Fisher's z) | -0.045 | -0.009 | — | |
| | SE Effect size | 0.015 | 0.015 | — | |
| 4. MD | Pearson's r | -0.007 | -0.002 | -0.011 | — |
| | p-value | 0.650 | 0.906 | 0.437 | — |
| | Effect size (Fisher's z) | -0.007 | -0.002 | -0.011 | — |
| | SE Effect size | 0.015 | 0.015 | 0.015 | — |

We performed a series of linear regressions with age and sex included as covariates. A whole brain analysis showed a negative relation between FA and solving CRA problems via insight ($R^2$ = .012; $p <$ .001); and a direct positive relation between MD and solving via insight ($R^2$ = .011; $p <$ .001). An overall analysis of 61 tracks (Bullock et al., 2022; Hanekamp et al., 2021) was performed; significant differences across the specific brain tracts are reported in Tables 2 and 3.

*Table 2 Tracts in which insight is significantly inversely related to FA and positively related to MD within regions in the left and right hemispheres. In each linear regression age, and sex were entered as covariates.*

| Insight | | | | - FA | | | + MD | | |
|---|---|---|---|---|---|---|---|---|---|
| Tract | MNI X | MNI Y | MNI Z | *p* | $R^2$ | F | *p* | $R^2$ | F |
| Left Posterior Arcuate Fasciculus | -37.6 | -49. 5 | 12.4 | .003 | .17 | 5.06 | .041 | .10 | 2.9 |
| Left Superior Longitudinal Fasciculus III (SLF III) | -3.8 | -15.9 | 19.9 | .023 | .12 | 3.3 | .027 | .11 | 3.2 |
| Right Superior Longitudinal Fasciculus III (SLF III) | 3.6 | -14.3 | 2 | .015 | .13 | 3.7 | .027 | .12 | 3.2 |

*Table 3 Tracts in which Step-by-step analysis was significantly positively related to FA and inversely with MD within regions in the left and right hemispheres. In each linear regression age, and sex were entered as covariates.*

| Step by Step | | | | | + FA | | | - MD | | |
|---|---|---|---|---|---|---|---|---|---|---|
| Tract | MNI X | MNI Y | MNI Z | Voxels | *p* | $R^2$ | F | *p* | $R^2$ | F |
| Anterior frontal Corpus Callosum + Forceps Minor | .9/0.6 | 3.19/3.4 | 6.1/6.8 | | < .001 | .1 | 5.5 | - | - | - |
| Left Vertical Occipital Fasciculus (VOF) | -17 | -8.1 | 4.9 | | .019 | .13 | 3.5 | .018 | .13 | 3.05 |

### 4.6 Age and education

We performed an overall correlation analysis between FA, MD, age, and years of education. Results are reported in Table 4. In line with prior studies, FA exhibited an age-related inverse correlation, due to myelin degradation, axonal loss, and increased extracellular water, leading to less restricted and more random water diffusion in white matter. As the brain ages, structural integrity declines, causing water molecules to diffuse in multiple directions rather than along well-organized pathways, which reduces FA values (Grieve et al., 2007). Higher education levels are often associated with higher FA, particularly in white matter regions related to cognitive function, such as the corpus callosum and prefrontal pathways. While FA naturally decreases with age, individuals with more education tend to show slower declines, possibly due to better-maintained brain networks which may explain why we find a milder negative correlation between FA and education in our dataset analysis (Teipel et al., 2009, 2010).

*Table 4. Correlation Matrix FA, MD, years of education and age.*

| Variable | | 1 | 2 | 3 | 4 |
|---|---|---|---|---|---|
| 1. FA | Pearson's r | — | | | |
| | p-value | — | | | |
| | Effect size (Fisher's z) | — | | | |
| | SE Effect size | — | | | |
| 2. MD | Pearson's r | -0.011 | — | | |
| | p-value | 0.437 | — | | |
| | Effect size (Fisher's z) | -0.011 | — | | |
| | SE Effect size | 0.015 | — | | |
| 3. Education | Pearson's r | -0.033 * | $-5.381\times10^{-4}$ | — | |
| | p-value | 0.026 | 0.971 | — | |
| | Effect size (Fisher's z) | -0.033 | $-5.381\times10^{-4}$ | — | |
| | SE Effect size | 0.015 | 0.015 | — | |
| 4. Age | Pearson's r | -0.043 ** | -0.009 | 0.521 *** | — |
| | p-value | 0.004 | 0.557 | < .001 | — |
| | Effect size (Fisher's z) | -0.043 | -0.009 | 0.577 | — |
| | SE Effect size | 0.015 | 0.015 | 0.015 | — |

* p < .05, ** p < .01, *** p < .001

[3] Average of problems solved per participant, calculated on the total number of given problems (Salvi et al., 2024).

## 5. Discussion

Insight problem solving is a distinct cognitive process essential for creative cognition. This study investigated the relationship between white matter microstructure and individual differences in insight and step-by-step analytical problem solving using DTI. Our findings reveal distinct white matter correlates for Aha! Moments, providing novel evidence for the structural basis of these cognitive processes. While prior research primarily focused on functional imaging, EEG, and brain stimulation (Kounios et al., 2006; Kounios & Beeman, 2014; Jung-Beeman et al., 2004; Salvi et al., 2020; Salvi, 2024), our results extend this understanding by uncovering structural foundations supporting insight problem solving. This bridges the gap between functional and structural results on idea generation via insight and creative cognition.

### 5.1 Insight-related findings

Our results indicate that insight propensity correlates with lower FA (matched by higher MD) in the left posterior AF, which connects the left STG (encompassing auditory cortex, angular gyrus, and Wernicke's area) to parietal regions. This pathway supports language processing, semantic integration, and phonological working memory—functions that may compete with the cognitive flexibility required for insight (Eichert et al., 2019). Further, lower FA (matched by higher MD) was found bilaterally in the Superior Longitudinal Fasciculus III (SLF III). This tract connects the TPJ (intersecting Wernicke's area) with the Inferior Frontal Gyrus (IFG, Broca's area in the left hemisphere). Notably, the left SLF III supports speech processing, while the right SLF III facilitates visuospatial functions (Janelle et al., 2022).

Together, lower FA in the left posterior AF and SLF III—connecting superior temporal, angular, post-central parietal, and inferior frontal cortices—suggests that strongly integrated language-related networks may inhibit insight. This aligns with findings from Shamay-Tsoory et al. (2011), who found that patients with left temporoparietal and inferior frontal lesions exhibited higher originality on divergent thinking tasks, implying a "releasing effect" on creativity. Similar effects have been observed in stroke (Mayseless et al., 2014) and frontotemporal dementia patients (Miller et al., 1996, 2000; Seeley et al., 2008), where lesions in left frontotemporal regions have been shown to enhance artistic creativity. Altogether, these findings imply that left-hemispheric regions play a regulatory role in creativity, and their disruption lifts this constraint, thus promoting novel ideas.

Research on hemispheric differences in semantic processing further supports this interpretation. Beeman et al. (1998; 2005) proposed that the left hemisphere engages in fine semantic coding—generating focused, context-specific semantic fields—while the right hemisphere performs coarse coding, integrating broader, loosely connected concepts (Chiarello et al., 1990). This broad integration facilitates unconventional associations, critical for insight (Kounios & Beeman, 2014). The sudden emergence of insight likely reflects a buildup of weakly activated solution-related information, reaching a threshold before bursting into consciousness — the classic Aha! Moment. Thus, reduced left-hemisphere structural connectivity supports theories suggesting that insight thrives on less constrained semantic processing and distant information integration. FA variations in these pathways may influence alpha-band oscillatory dynamics, modulating selective inhibition mechanisms crucial for insight. This aligns with prior evidence linking alpha waves to insight's unconscious buildup phase, where suppressed dominant strategies allow for novel connections between concepts to emerge in memory (Kounios et al., 2006). FA, a marker of white matter integrity, may thus explain individual differences in insight performance, linking structural properties to cognitive flexibility and creative ideation.

### 5.2 Analytical problem-solving findings

In contrast to insight, step-by-step analytical problem solving was positively associated with higher FA in the anterior CC and Forceps Minor and with higher FA (and lower MD) over the left VOF. These structural patterns likely support the more deliberate, executive-driven nature of step-by-step analytical problem solving. The CC is a key white matter structure that facilitates interhemispheric communication and integration of information between the left and right cerebral hemispheres (Gazzaniga, 2000). The stronger structural integrity of the anterior CC, which connects the prefrontal cortices, may enable the enhanced inter-hemispheric exchange of information required for the controlled, step-by-step approach characteristic of analytical problem-solving (Luders et al., 2010; Takeuchi et al., 2010, 2020). This increased callosal connectivity could support the coordination of executive functions, such as cognitive control, working memory, and attention, that are heavily recruited during analytical reasoning (Braver et al., 1995; Goel & Vartanian, 2005). Based on the existing literature by Takeuchi and coworkers' we predicted a positive association between FA and insight problem solving. However, it needs to be noted that none of those studies assessed how participants generated ideas (via insight or step-by-step analysis), thus we can speculate that the results obtained by Takeuchi and coworkers could be caused by a higher rate of unreported solutions via the step-by-step analysis, perhaps due to the nature of the task. Furthermore, the positive association between FA in the left VOF and step-by-step analytical problem solving is noteworthy (considering also the negative correlation with MD). The VOF is a ventral white matter tract (posterior to the AF and lateral to the optic radiation) that connects the dorsal and ventral visual cortex and is thought to be involved in spatial and object processing, as well as stereoacuity (Yeatman et al., 2013; Takemura et al., 2016; Oishi et al., 2018). Of note, the VOF is unique to primates' brains, as an adaptation that likely facilitated the evolution of visually guided behaviors and problem-solving (Takemura et al., 2024). The left VOF has also been linked to the identification of written words, involved in grapheme-phoneme conversion and lexical access(Bouhali et al., 2014) Higher FA in the left VOF, suggests more efficient information transfer between visual processing areas and higher cognitive regions, may indicate enhanced analytical problem-solving abilities, particularly in visual-spatial and language-related tasks. The enhanced structural integrity of this pathway may facilitate the integration of word-related perceptual information with goal-directed, top-down processing required for the step-by-step approach that characterizes analytical problem solving (Chrysikou & Thompson-Schill, 2011; Gonen-Yaacovi et al., 2013). The left lateralization of this effect between FA in the VOF and analytical problem solving aligns with the left hemisphere's role in fine semantic coding and focused cognitive control, in contrast with the right hemisphere's broader, more diffuse semantic processing that appears to support insight (Jung-beeman, 2005; Subramaniam et al., 2009).

### 5.3 Comparison with previous DTI studies on creativity

Our findings partially align with, yet also diverge from, previous DTI studies on creative cognition. For instance, prior studies (Jung et al., 2010; Wertz et al., 2020) reported lower levels of FA within left inferior frontal tracts (overlapping the uncinate fasciculus and anterior thalamic radiation) and divergent thinking (measured by a Creative Composite Index). In our study, we found a similar inverse relationship between FA and insight within the SLF III that projects to the left Inferior Frontal Lobe, corroborating the idea that left IFG damage may produce a "releasing effect" on creativity, allowing for more novel and unconventional ideas which might rise as sudden insight. This relationship thus points to an overlap between divergent thinking and insight, rather than analytical problem-solving. Nonetheless, insightfulness is not usually measured in divergent thinking tasks leaving this last conclusion so far just a speculation. By contrast, Takeuchi et al. (2010, 2020) reported positive correlations between FA and both divergent and convergent thinking abilities across several bilateral white matter tracts (convergent thinking - left ILF. and left frontal-occipital fasciculus; divergent thinking - CC, the bilateral basal ganglia, the bilateral TPJ, and the right inferior parietal lobe). Such findings broadly align with our observation that analytical problem solving is positively associated with FA across the frontal CC and the VOF. As such, it is possible that the findings by Takeuchi and colleagues (2010) were driven by a higher rate of problems being solved via step-by-step analysis. However, the work by Takeuchi and colleagues (2010)

did not assess how participants generated ideas (via insight or step-by-step analysis), thus leaving this question unanswered.

Past studies linking FA and creative cognition only measured task performance, in terms of either the originality of ideas (in divergent thinking) or the number of problems that were solved correctly (in convergent thinking tasks). Insight problem-solving can potentially occur not only in convergent tasks like the CRA but also during divergent thinking, however, this has rarely been investigated in past literature. Consequently, given the general lack of research on the neuroscience of insight for divergent thinking tasks, it remains unclear whether our conclusions can be directly extended to divergent thinking performance.

In sum, our study addresses a significant gap in the literature by directly comparing the structural correlates of insight and analytical problem-solving. The present investigation enables us to disentangle the specific neural architecture supporting the distinct cognitive processes of insight and step-by-step analysis, a differentiation that was not addressed in past studies. Our findings support and extend previous research on the neural basis of creative cognition. The lower FA associated with insight in several tracts is consistent with some previous studies on divergent thinking (Jung et al., 2010; Wertz et al., 2020), suggesting potential overlaps between insight and broader aspects of creative cognition. The positive relationship between FA and step-by-step analytical problem solving in several tracts aligns more closely with previous research on convergent thinking (Rahmani et al., 2020; Takeuchi et al., 2020). This association suggests that the effects observed in such work could be related to the influence of analytical processing.

Discrepancies between our findings and those of previous studies underscore the complexity of creative cognition, and the importance of specificity and standardized methodological approaches when investigating idea generation in neuroimaging research. As such, future studies should continue to refine the operational definitions and measurements of unique creative processes to better understand their unique and shared neural substrates.

## 6. Conclusion

Our study provides novel evidence for distinct structural connectivity patterns associated with insight and analytical problem-solving. The findings suggest that insight is associated with lower FA in several left hemisphere tracts, including the posterior AF, and bilateral SLF III. These results complement the established literature documenting relationships between FA and cognitive functions (Johansen-Berg, 2010; Mori & Zhang, 2006). Specifically, lower FA across left hemisphere tracts reflects a more diffuse connectivity pattern that may allow for broader semantic activation and cognitive flexibility necessary for insight (Kounios & Beeman, 2014; Beeman & Bowden, 2000). The involvement of the SLF III, which connects frontal, temporal, and parietal regions (Catani & Thiebaut de Schotten, 2008; Dick & Tremblay, 2012), suggests that insight may rely on more distributed neural activation rather than highly focused connections (Jung-Beeman et al., 2004; Subramaniam et al., 2009). This pattern of structural connectivity aligns with functional neuroimaging studies that have highlighted the importance of widespread activation in insight problem solving (Jung-Beeman et al., 2004).

In contrast, analytical problem-solving was associated with higher FA in the anterior CC. This pattern indicates that step-by-step problem-solving may benefit from stronger, more directed structural connections, particularly in pathways involving frontal and thalamic regions crucial for executive control and deliberate cognitive processing (Miller & Cohen, 2001; Bunge et al., 2005). The dissociation between insight and analytical problem-solving is particularly noteworthy, highlighting the distinct neural architectures supporting these two modes of problem-solving. These results contribute to a more nuanced understanding of the structural basis underlying different aspects of idea generation in creative cognition. Our findings pave the way for future investigations into how variations in white matter microstructure may influence an individual's propensity for insight versus analytical thinking, and how these structural differences relate to functional activation patterns observed during creative cognition.

### Limitations and future directions

While our study provides valuable insights into the structural correlates of insight and step-by-step analytical problem-solving, some limitations should be noted. First, the cross-sectional nature of our study precludes causal inferences about the relationship between white matter structure and problem-solving abilities. Longitudinal studies or training interventions could help clarify the directionality of these relationships. On a similar note, integrating DTI data with functional neuroimaging and electrophysiological measures would offer a more complete understanding of how structural connectivity supports the dynamic neural processes involved in insight and analytical problem solving. As such, future work should extend the present study by employing a more multimodal methodology, thus providing a more complete picture of the neuroscience of insight.

It should then be noted that although we controlled for age and sex in our analysis, the demographic characteristics of our sample, such as socioeconomic status, and education, might have all affected the observed results. Indeed, past work indicated that negative correlations between FA and divergent thinking across several tracts were only observed for female participants, while males exhibited the opposite effect (Ryman et al., 2014; Takeuchi et al., 2017). Such work outlines the importance of considering sample characteristics such as gender when investigating links between individual differences in creative cognition and patterns in white matter integrity. Nevertheless, it is worth noting that the present sample was roughly balanced between male and female participants. While no work to date has reported interactions between creative abilities and age, socioeconomic status, or educational level, all of these variables have been noted to influence white matter integrity (Penke et al., 2010; Nobel et al., 2013; Shakel et al., 2009 Teipel et al., 2009; Vernooij et al., 2009).

Of note, our findings may not extend to all other convergent thinking tasks. Indeed, solving anagrams via insight has been shown to elicit left-lateralized brain activity during insight (Oh et al., 2020). This finding has been attributed to the unique task demands of completing anagrams, compared to other insight problems such as the CRA. While anagram completion relies heavily on grapheme feature integration, the CRA uniquely requires the integration of semantically distant concepts. Our decision to employ the CRA in the present study was guided by current best practices in insight research and to coherence with prior imaging studies (i.e., Jung-Bemman et al., 2004).

The study of insight has undergone a significant methodological shift, transitioning from classic problems (Duncker, 1945; Maier, 1930) to newer paradigms like the CRA (Bowden & Jung-Beeman, 2003b; MacGregor & Cunningham, 2008).  This change was driven by the need for increased statistical power and neuroimaging compatibility which is afforded by the CRA (Bowden et al., 2005; Ludmer et al., 2011; Salvi et al., 2015; 2020). Traditional insight problems, such as the nine-dot or two-string problems (Duncker, 1945; Maier, 1930), while directly tapping into insight processing, presented limitations in terms of solving reliability and adaptability to neuroscientific methods. However, this methodological shift has led to a continued focus on convergent thinking tasks to measure insight, potentially overlooking how insightful ideas emerge during divergent thinking processes. Insight, which is fundamentally about idea generation, may manifest in both convergent and divergent thinking scenarios, suggesting the need for a more comprehensive approach to studying this phenomenon. As such, future work is required to determine whether the present findings extend to insight into other convergent thinking tasks, or even divergent thinking studies.

## Declarations

#### Author Declarations

#### Ethics approval

This study was approved by the Institutional Review Board of Northwestern University.

#### Consent to participate

All participants provided written informed consent prior to participation in the study.

#### Consent for publication

Not applicable.

#### Data Availability

The datasets generated and/or analyzed during the current study are available on brainlife.app.

#### Competing interests

The authors declare that they have no competing interests.

#### Funding

This research was supported by the United States Air Force Research Laboratory FA8650-15-2-5518 to MB, and by the Smart Family Foundation of New York to JG. CS was supported in part by NIH training grant T32 NS047987.

**Acknowledgements**

We thank the participants for their time and commitment to the study. We also acknowledge the support provided by the Center for Translational Imaging at Northwestern University and the developers and maintainers of the Brainlife.app platform.

**Clinical Trial**

Clinical trial number: Not applicable.

## Figures

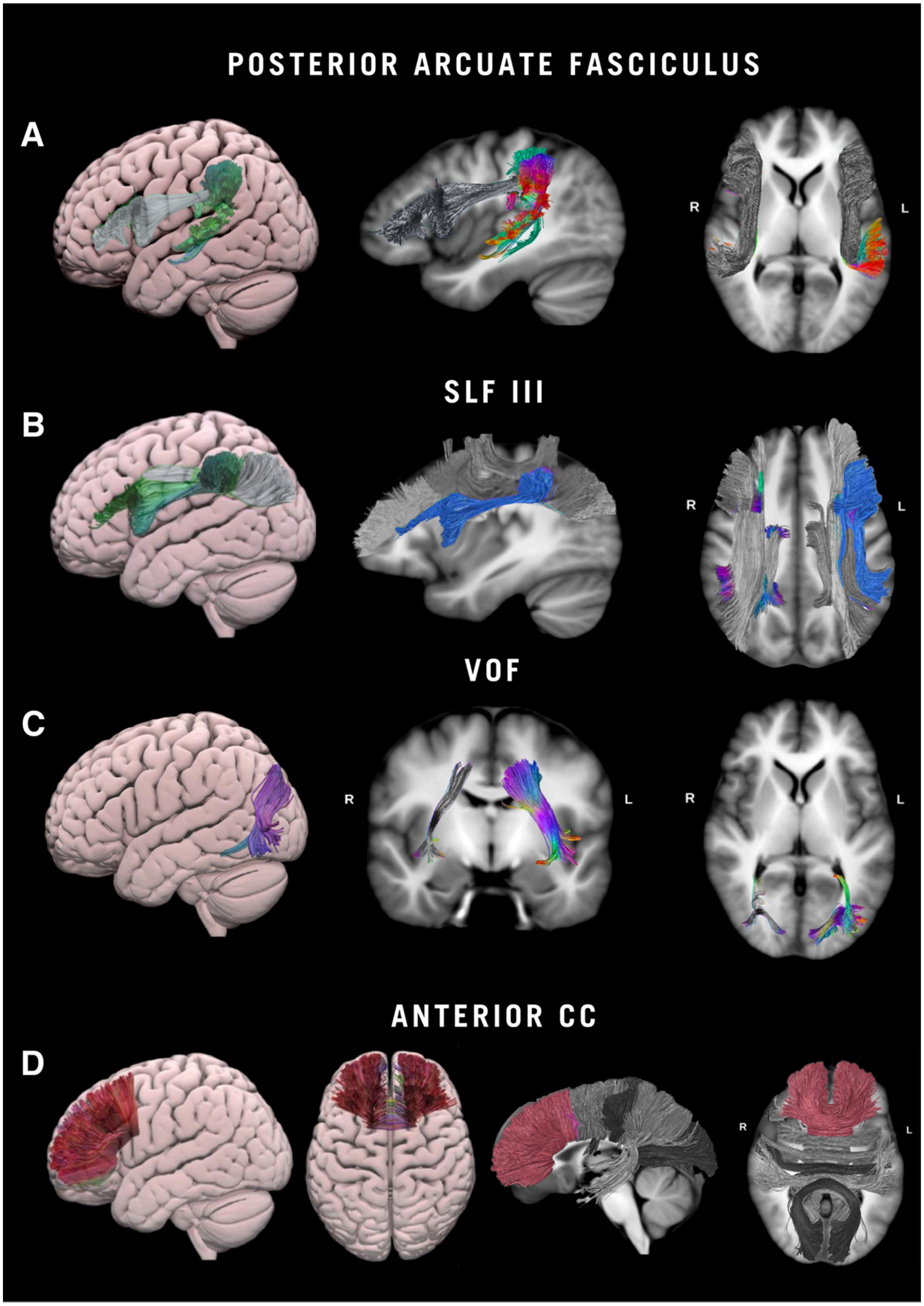

### Figure 1

*A. From left to right: 3D lateral projection of the left posterior Arcuate Fasciculus (AF) in green overlaid on semitransparent MNI pial surface. Left  posterior AF overlaid in directional color coding on T1-weighted images.*

*B. From left to right: 3D lateral projection of the Superior Longitudinal Fasciculus III (SLF III) in green overlaid on semitransparent MNI pial surface. Left SLF III in blue overlaid in directional color coding on T1-weighted images.*

*C. From left to right: 3D lateral projection of the Vertical Occipital Fasciculus (VOF) in purple overlaid on semitransparent MNI pial surface. Left SLF VOF in blue and purple overlaid in directional color coding on T1-weighted images.*

*D. From left to right: 3D lateral and superior projection of the Anterior Frontal Corpus Callosum and Forceps Minor in red overlaid on semitransparent MNI pial surface. Lateral and superior projection of the Anterior Frontal Corpus Callosum (CC) and Forceps Major and red overlaid in directional color coding on T1-weighted images. Atlas taken from: Radwan et al., 2021.*

### Supplementary Files

This is a list of supplementary files associated with this preprint. Click to download.

- SupplementaryMaterial.docx
